# Supplementary material for: Limbic System Response to Psilocybin and Ketamine Administration in Rats: A Neurochemical and Behavioral Study
Source: Int J Mol Sci. 2023 Dec 20;25(1):100. doi: 10.3390/ijms25010100 (PMC10779066; doi:10.3390/ijms25010100)

On each blot 2 animals (one cohort) were used from 8 different cohorts

**A blot**

| | |
|---|---|
| **Control** | **1, 2** |
| **Psilocybin 2mg/kg** | **1, 2** |
| **Psilocybin 10mg/kg** | **1, 2** |
| **Ketamine 10mg/kg** | **1, 2** |

**B blot**

| | |
|---|---|
| **Control** | **3, 4** |
| **Psilocybin 2mg/kg** | **3, 4** |
| **Psilocybin 10mg/kg** | **3, 4** |
| **Ketamine 10mg/kg** | **3, 4** |

**C blot**

| | |
|---|---|
| **Control** | **5, 6** |
| **Psilocybin 2mg/kg** | **5, 6** |
| **Psilocybin 10mg/kg** | **5, 6** |
| **Ketamine 10mg/kg** | **5, 6** |

**D blot**

| | |
|---|---|
| **Control** | **7, 8** |
| **Psilocybin 2mg/kg** | **7, 8** |
| **Psilocybin 10mg/kg** | **7, 8** |
| **Ketamine 10mg/kg** | **7, 8** |

**N=8**

# HIPPOCAMPUS

## 5HT1A

### A blot (this blot in publication)

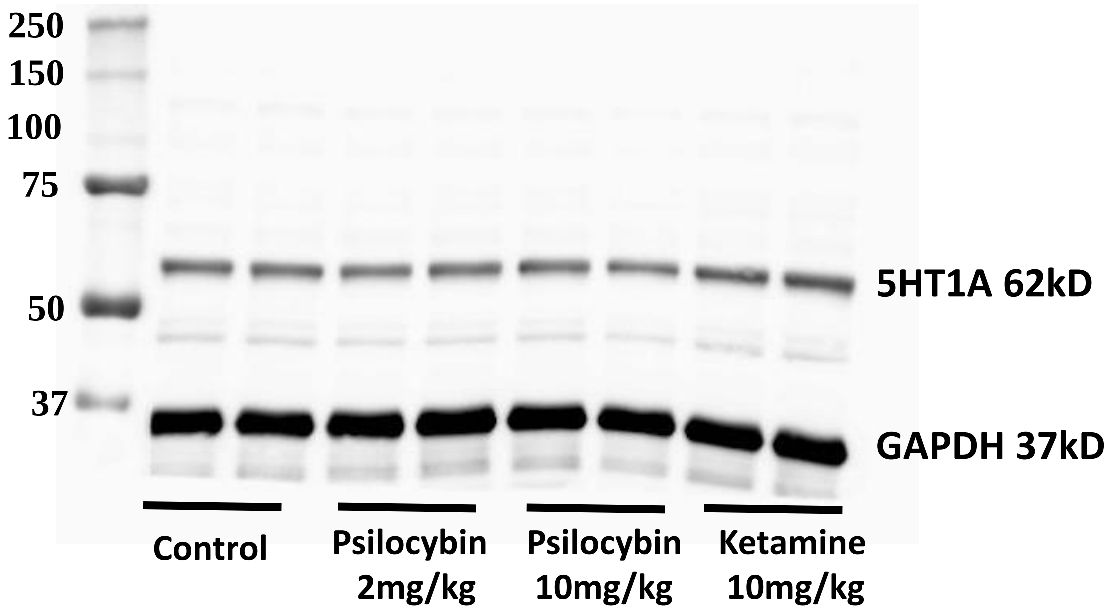


### B blot

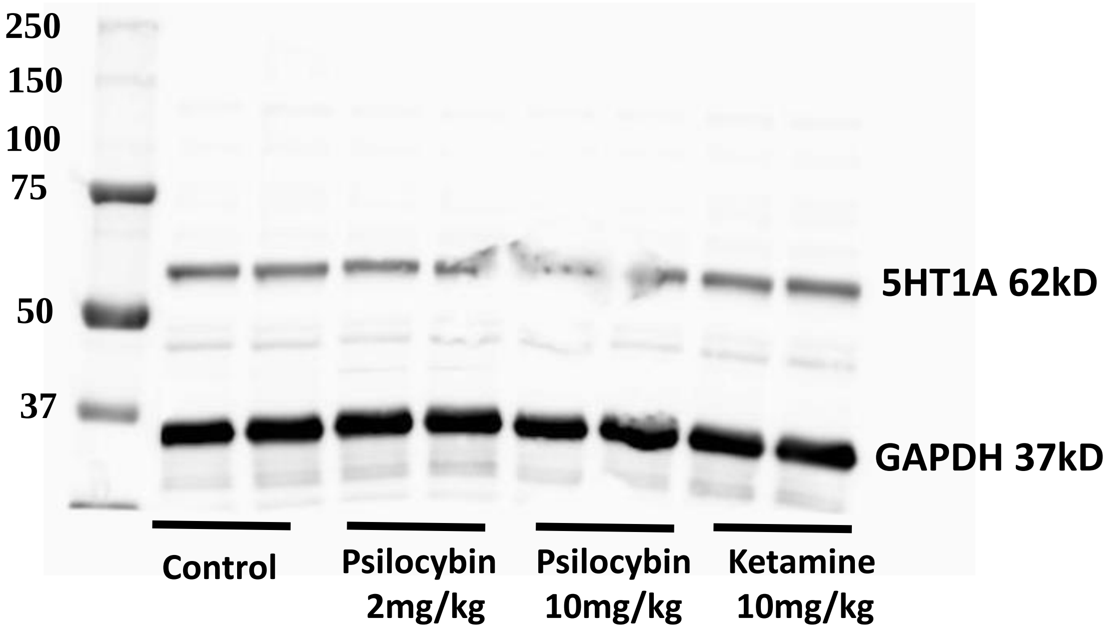

# HIPPOCAMPUS

## 5HT1A

### C blot

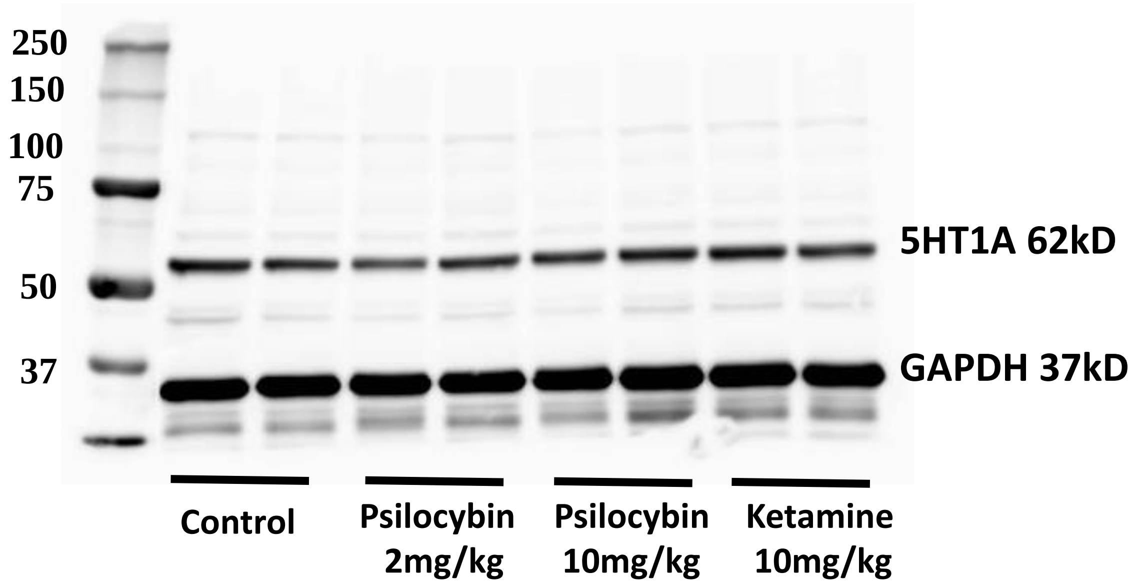


### D blot

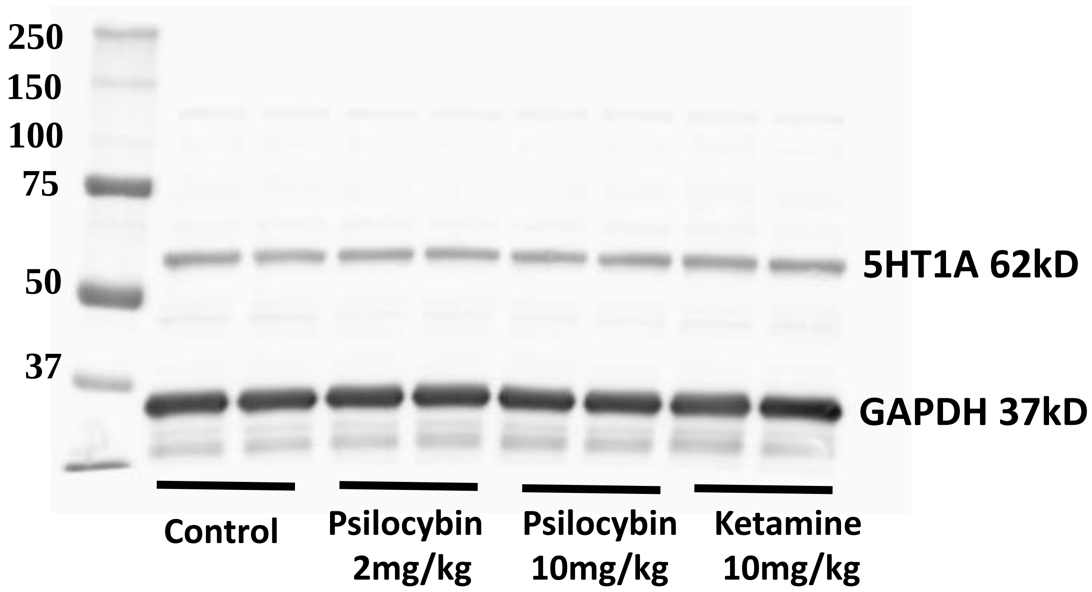

# HIPPOCAMPUS

## 5HT2A

**A blot** **(this blot in publication)**

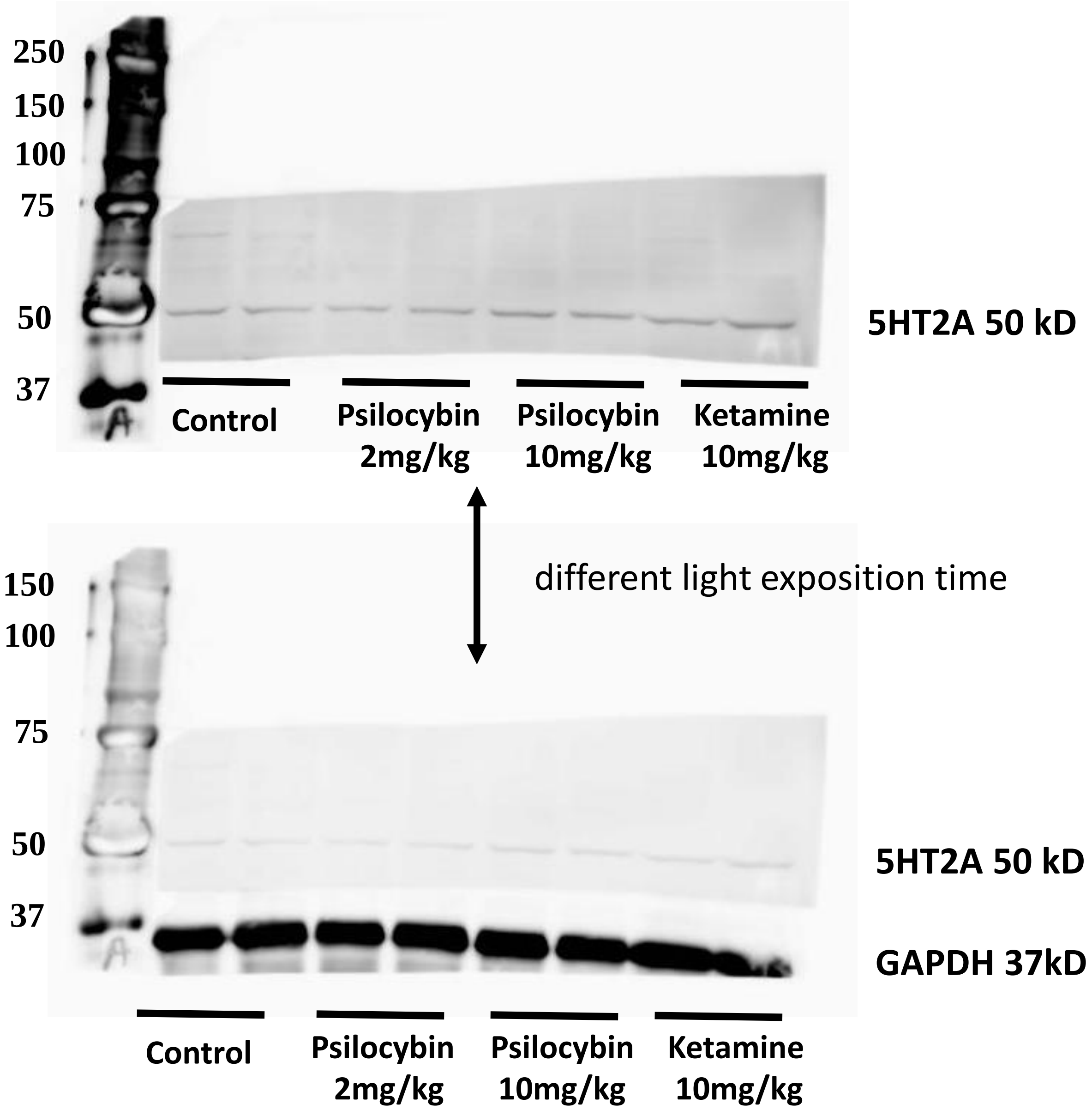


**B blot**

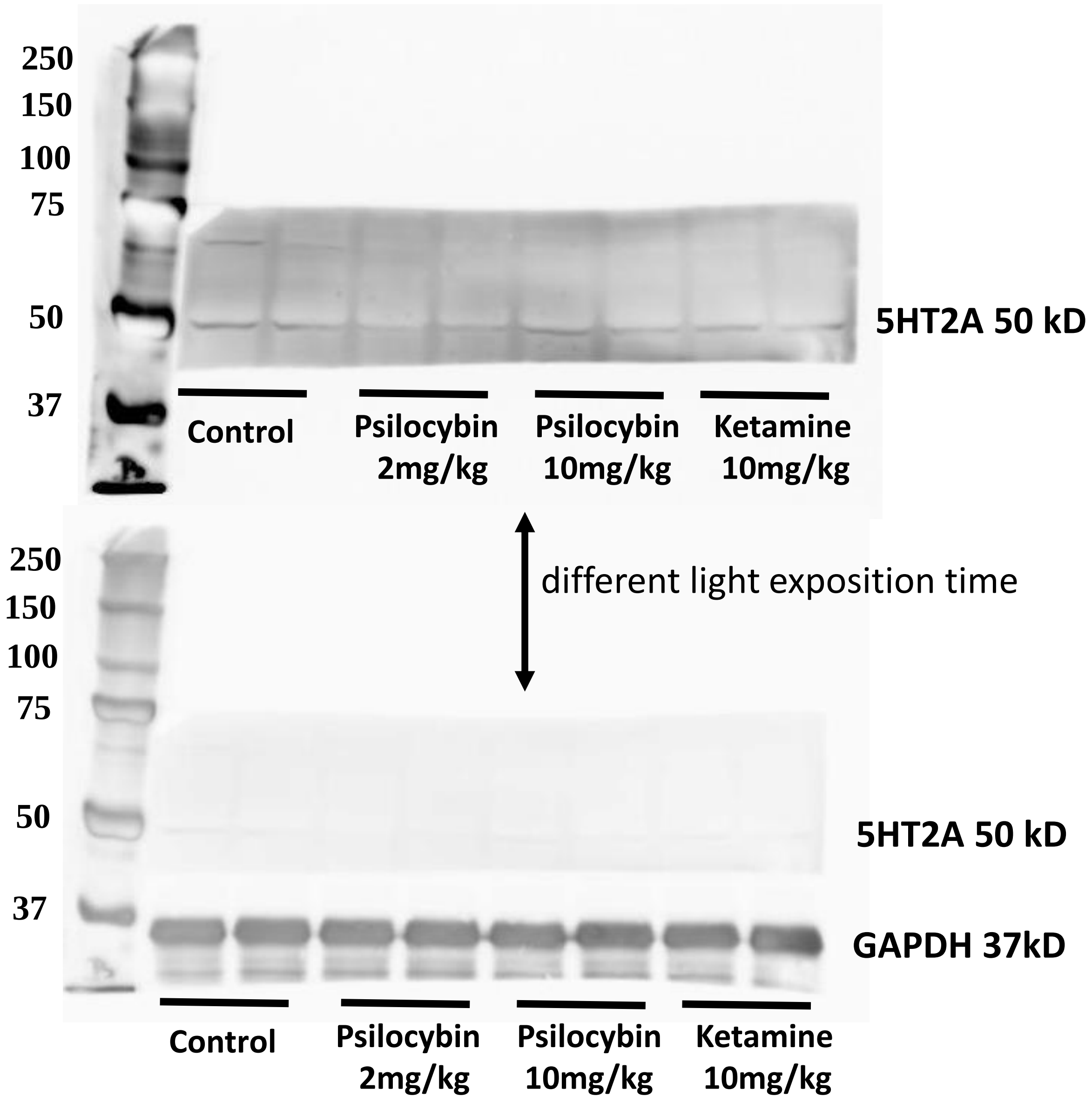

# 5HT2A HIPPOCAMPUS

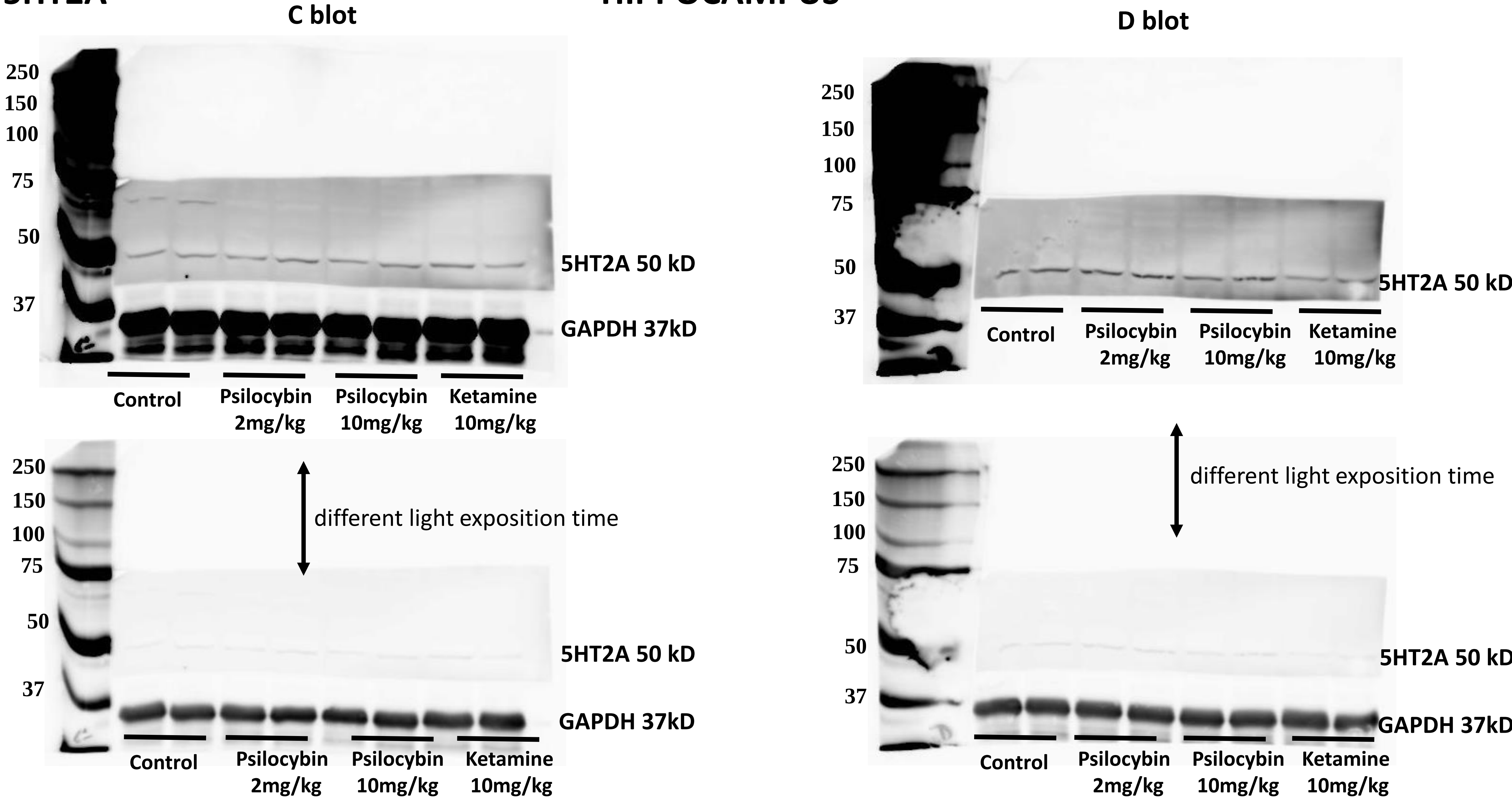

# Nucleus Accumbens

## 5HT2A

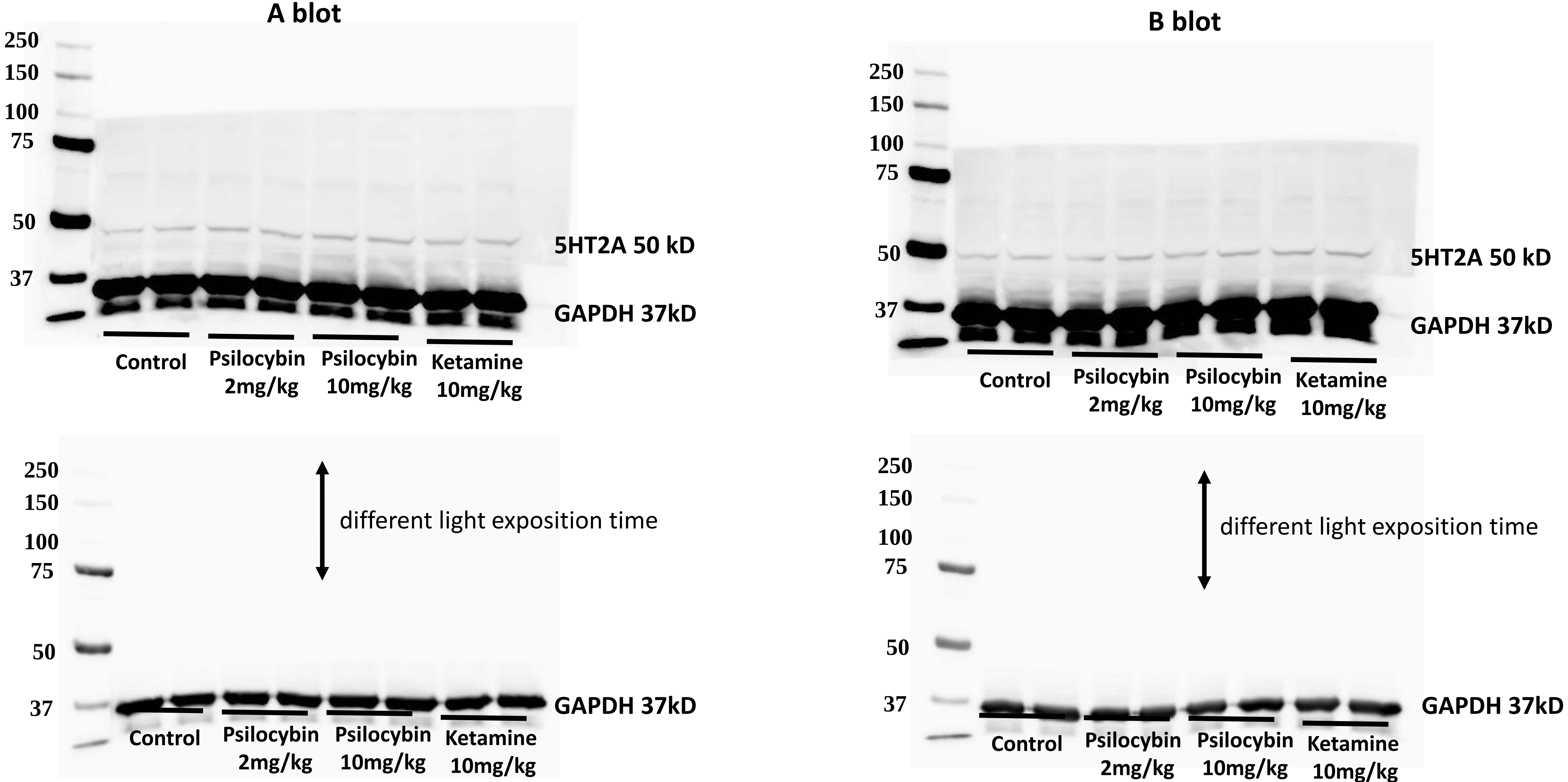

# Nucleus Accumbens

## 5HT2A

### C blot

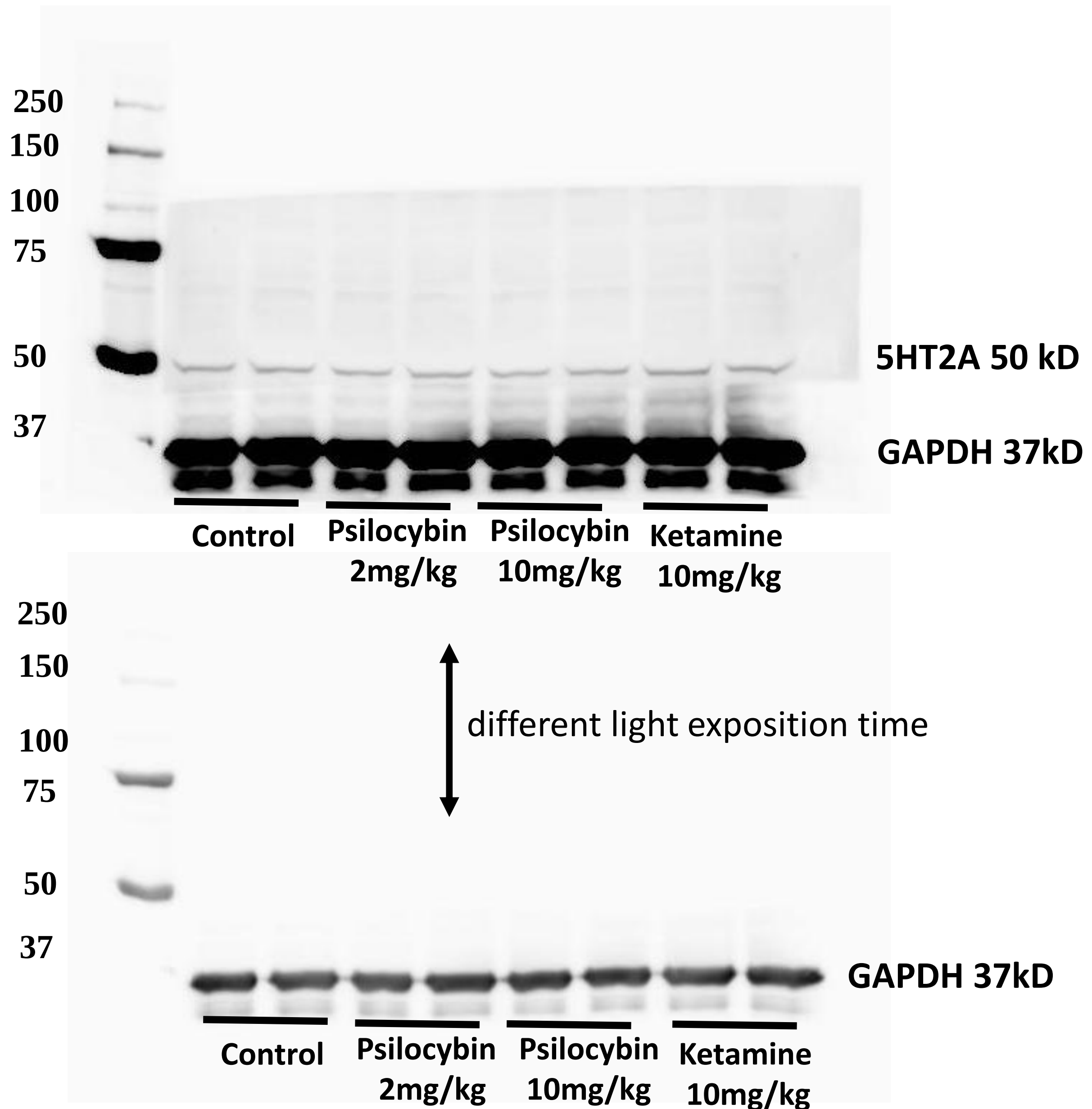


### D blot (this blot in publication)

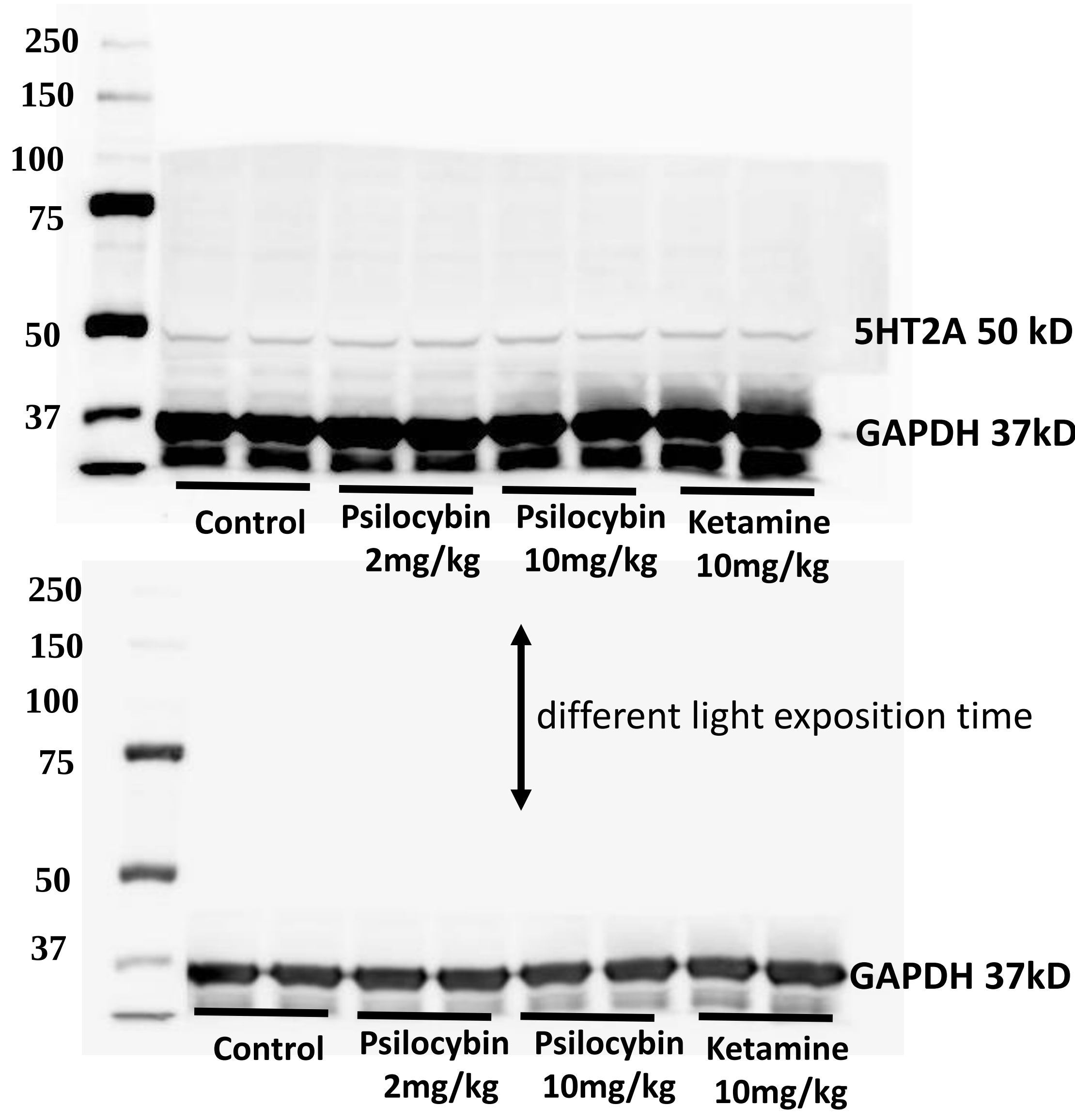

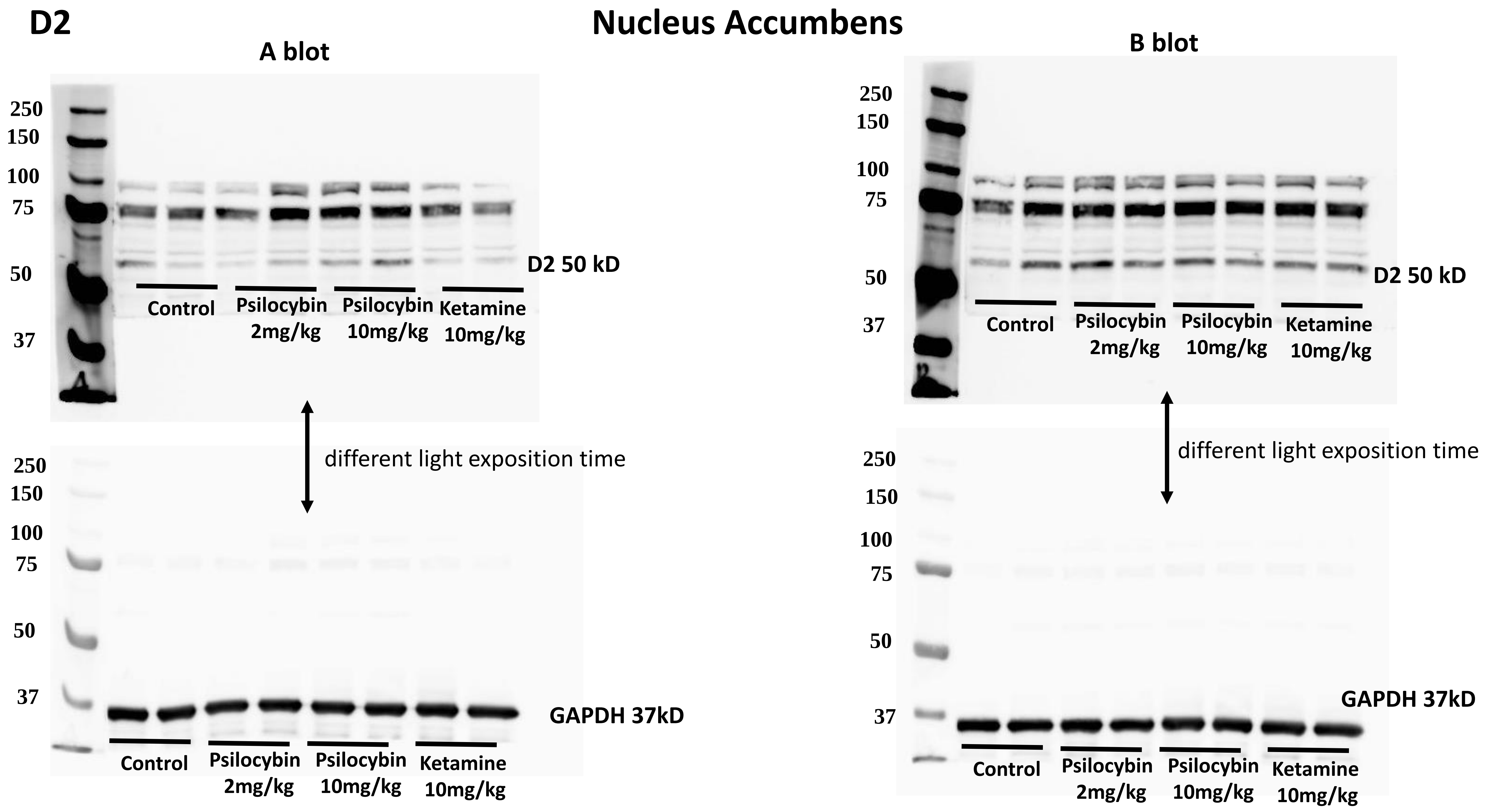
D2
Nucleus Accumbens
A blot
250
150
100
75
50
37
D2 50 kD
Control
Psilocybin 2mg/kg
Psilocybin 10mg/kg
Ketamine 10mg/kg
different light exposition time
250
150
100
75
50
37
GAPDH 37kD
Control
Psilocybin 2mg/kg
Psilocybin 10mg/kg
Ketamine 10mg/kg
B blot
250
150
100
75
50
37
D2 50 kD
Control
Psilocybin 2mg/kg
Psilocybin 10mg/kg
Ketamine 10mg/kg
different light exposition time
250
150
100
75
50
37
GAPDH 37kD
Control
Psilocybin 2mg/kg
Psilocybin 10mg/kg
Ketamine 10mg/kg

# Nucleus Accumbens

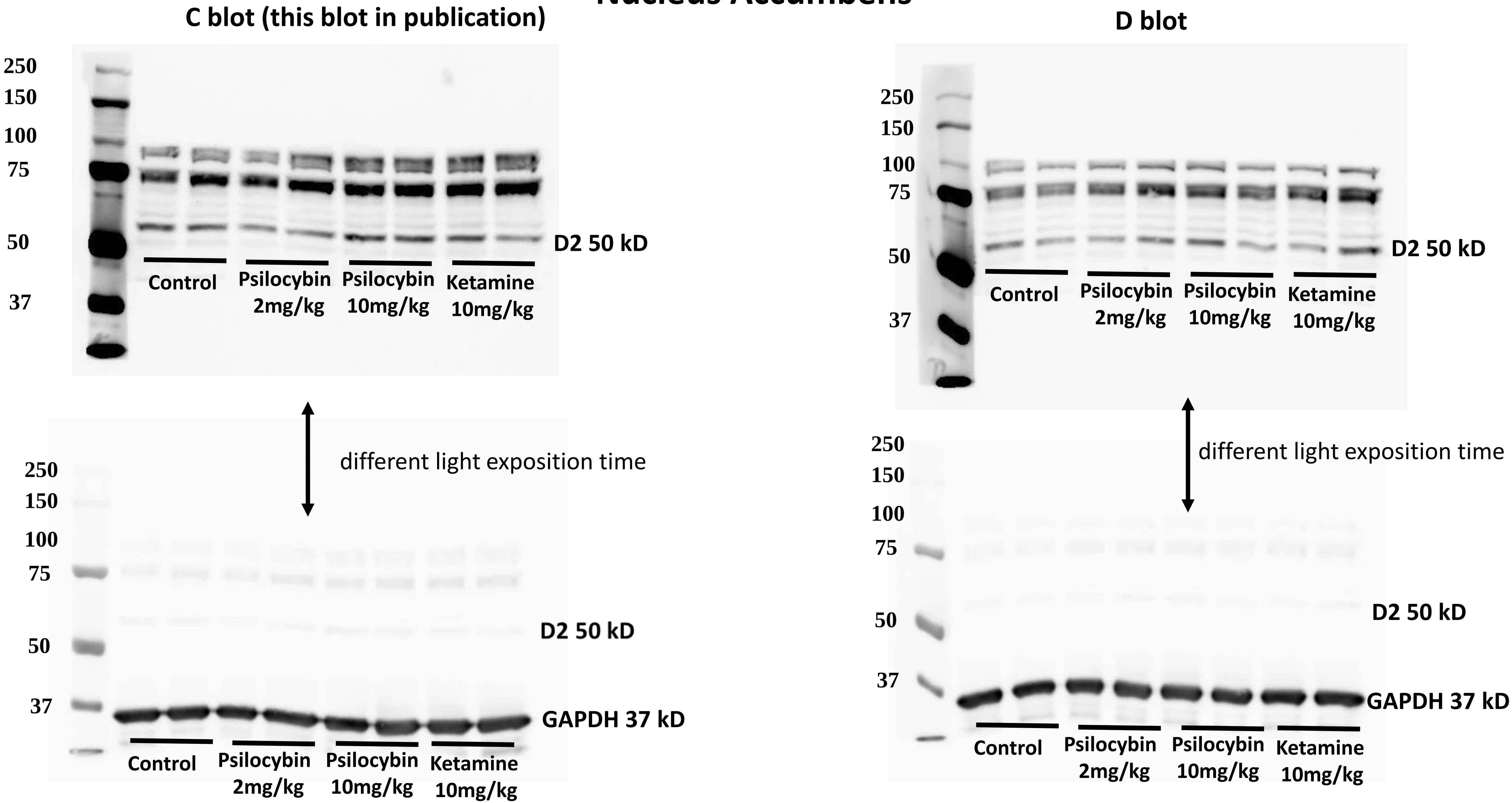

Supplement: Supplementary file 1 [file ijms-25-00100-s001.zip › ijms-2650891-supplementary/Supplementary Materials File S1.pdf]
