# Supplementary material for: Limbic System Response to Psilocybin and Ketamine Administration in Rats: A Neurochemical and Behavioral Study
Source: Int J Mol Sci. 2023 Dec 20;25(1):100. doi: 10.3390/ijms25010100 (PMC10779066; doi:10.3390/ijms25010100)
Supplement: Supplementary file 1 [file ijms-25-00100-s001.zip › ijms-2650891-supplementary/Supplementary Materials File S2.pdf]

Supplementary Materials File S2

Method

Elevated plus maze. The apparatus comprised two open (50× 11 cm) and two non-transparent closed (50× 11 × 40 cm) arms elevated 50 cm above the floor and illuminated by two 25 W bulbs located beneath the open arms. The animals were placed individually in the center of the apparatus and the number of entries of all 4 feet into open and closed arms, and the time spent in each arm, were recorded manually in a 5-min test by an observer who was blinded to the treatments. The results are expressed as a percentage of entries/ time in the open arms: open/(open+closed)× 100.

Results

The effect of psilocybin and ketamine on the activity of rats in the elevated plus maze

There was no effect of both doses of psilocybin or ketamine on open arm entries and time spent in open arms at 2 h ($F_{3,36} = 2.03$, $p < 0.126$, $F_{3,36} = 0.72$, $p < 0.55$, respectively, one-way ANOVA) or 24 h after drugs administration ($F_{3,36} = 1.26$, $p < 0.30$, $F_{3,36} = 1.86$, $p < 0.15$, respectively, one-way ANOVA) (Fig. 1S).

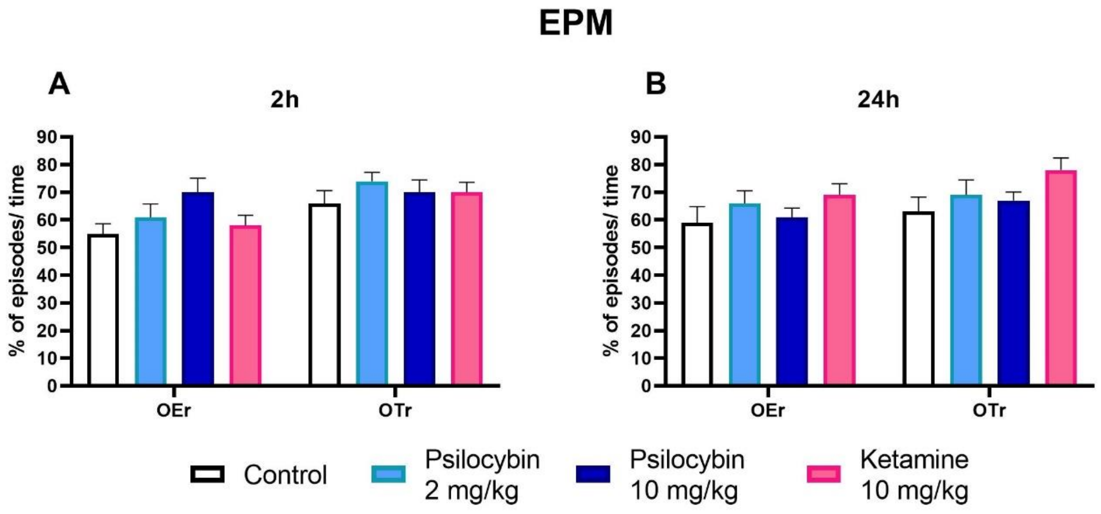

Figure S1. Open arm entries (OEr) and open arm time (OTr) shown as a percentage of total open + closed entries/time) in the elevated plus maze for control (white bars), psilocybin (2 and 10 mg/kg) and ketamine (10 mg/kg) 2 h (**A**) and 24 h (**B**) after administration. Values are the mean ± SEM (n = 10). One-way ANOVA followed by Tukey’s post hoc test.
