# Supplementary material for: Limbic System Response to Psilocybin and Ketamine Administration in Rats: A Neurochemical and Behavioral Study
Source: Int J Mol Sci. 2023 Dec 20;25(1):100. doi: 10.3390/ijms25010100 (PMC10779066; doi:10.3390/ijms25010100)
Supplement: Supplementary file 1 [file ijms-25-00100-s001.zip › ijms-2650891-supplementary/Supplementary Materials File S3.pdf]

Supplementary Materials File S3

Table S1. Basal levels of neurotransmitters in treatment groups

Treatment	DA (pg/10 µl)	5-HT (pg/10 µl)	GLU (ng/10 µl)	GABA (pg/10 µl)	ACh (nM)
**Nucleus accumbens**					
control	0.89 ± 0.05	0.26 ± 0.02	7.82 ± 0.83	43.36 ± 2.43	nd
Psilocybin 2 mg/kg	0.86 ±- 0.09	0.26 ± 0.02	7.72 ± 0.88	47.95 ± 4.99	nd
Psilocybin 10 mg/kg	0.85 ± 0.09	0.29 ± 0.03	7.70 ± 1.41	39.2 ± 2.26	nd
Ketamine 10 mg/kg	0.99 ± 0.04	0.22 ± 0.02	8.21 ± 0.81	44. 68 ± 5.33	nd
**Hippocampus**					
control	nd	nd	5.54 ± 0.41	42.97 ± 2.05	0.37 ± 0.06
Psilocybin 2 mg/kg	nd	nd	5.34 ± 0.62	35.89 ± 2.77	0.30 ± 0.09
Psilocybin 10 mg/kg	nd	nd	6.60 ± 0.49	49.72 ± 2.98	0.37 ± 0.16
Ketamine 10 mg/kg	nd	nd	4.77 ± 0.50	45.98 ± 3.79	0.43 ± 0.06
**Amygdala**					
control	nd	nd	4.38 ± 0.30	48 ± 2.82	nd
Psilocybin 2 mg/kg	nd	nd	4.77 ±3 0.51	44.5 ± 3.31	nd
Psilocybin 10 mg/kg	nd	nd	4.19 ± 0.48	49.0 ± 5.73	nd
Ketamine 10 mg/kg	nd	nd	4.08 ± 0.57	52.0 ± 4.9	nd
