# Supplementary figures and images for: Limbic System Response to Psilocybin and Ketamine Administration in Rats: A Neurochemical and Behavioral Study
Source: Int J Mol Sci. 2023 Dec 20;25(1):100. doi: 10.3390/ijms25010100 (PMC10779066; doi:10.3390/ijms25010100)

## NUCLEUS ACCUMBENS

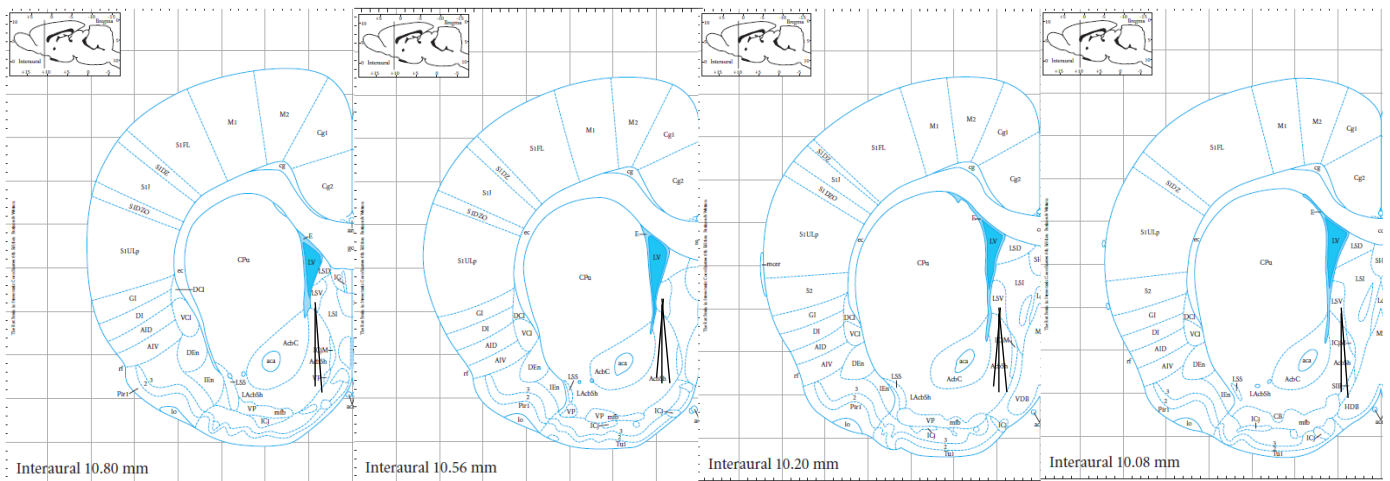

## AMYGDALA

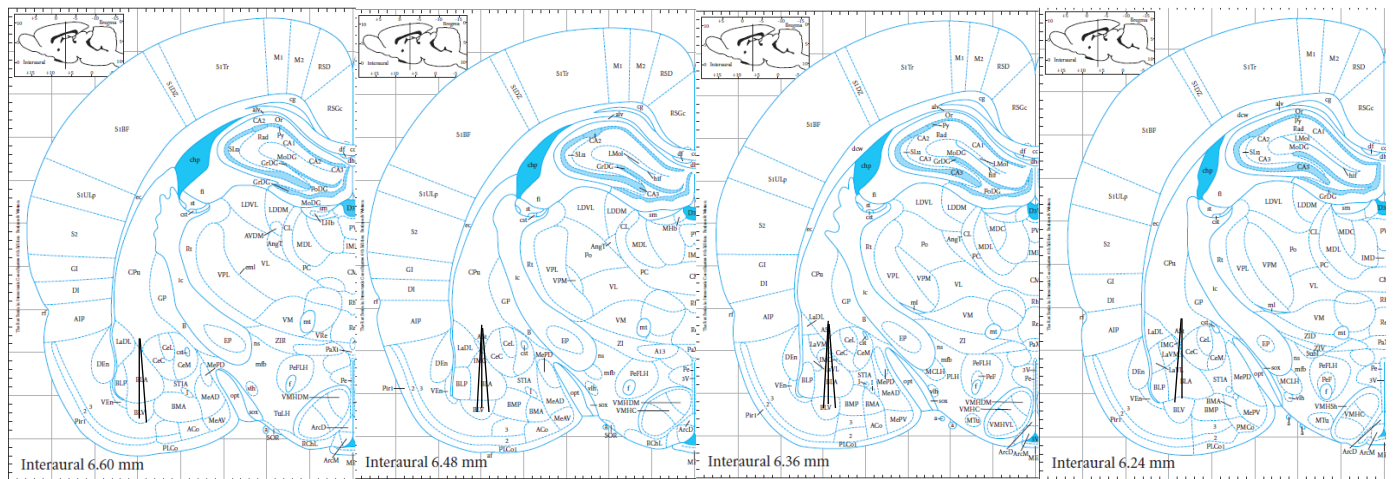

## HIPPOCAMPUS

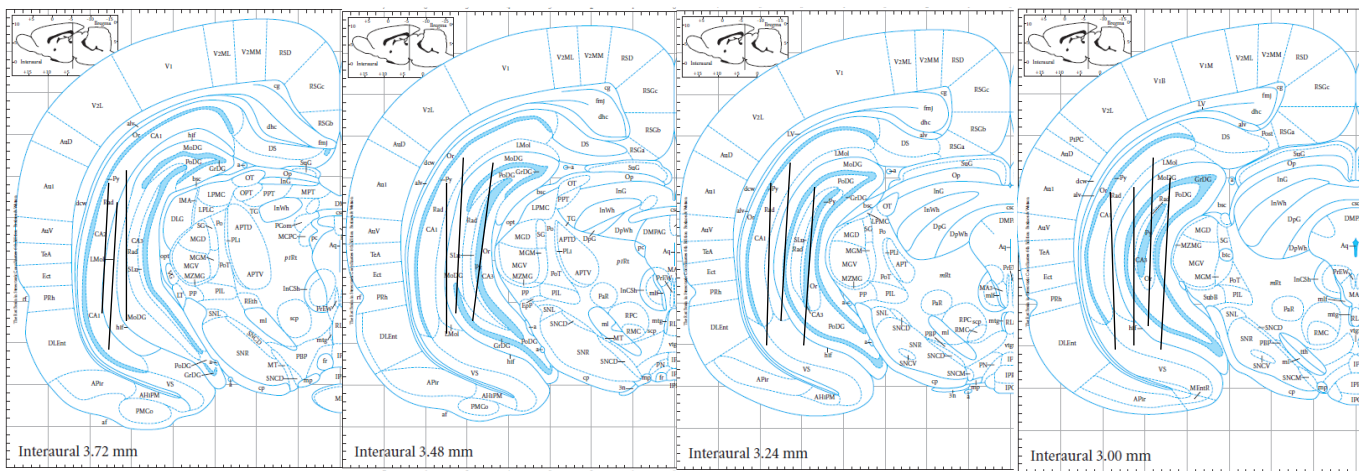

Supplement: Supplementary file 1 [file ijms-25-00100-s001.zip › ijms-2650891-supplementary/Supplementary Materials File S4.pdf]
